# Association Between Malnutrition, Low Muscle Mass, Elevated NT-ProBNP Levels, and Mortality in Hemodialysis Patients

**DOI:** 10.3390/nu17111896

**Published:** 2025-05-31

**Authors:** Sadamu Takahashi, Tatsuki Tanaka, Yusuke Suzuki, Norihito Yoshida, Mai Hitaka, Shingo Ishii, Keisuke Yamazaki, Motoyuki Masai, Yosuke Yamada, Yasushi Ohashi

**Affiliations:** 1 Department of Nephrology, Toho University Sakura Medical Center, Chiba 285-8741, Japan; sadamu.takahashi@med.toho-u.ac.jp (S.T.); tatsuki.tanaka@med.toho-u.ac.jp (T.T.); yusuke.suzuki@med.toho-u.ac.jp (Y.S.); norihito.yoshida@med.toho-u.ac.jp (N.Y.); mai.hitaka@med.toho-u.ac.jp (M.H.); shingo.ishii@med.toho-u.ac.jp (S.I.); keisuke.yamazaki@med.toho-u.ac.jp (K.Y.); 2 Department of Urology, Mihama Hospital, Chiba 261-0013, Japan; m.masai@seijinkai.org; 3 Department of Sports and Health Sciences, Graduate School of Biomedical Engineering, Tohoku University, Miyagi 980-8575, Japan; yosuke.yamada.c1@tohoku.ac.jp; 4 Department of Medicine and Science in Sports and Exercise, Graduate School of Medicine, Tohoku University, Miyagi 980-8575, Japan

**Keywords:** hemodialysis, malnutrition, sarcopenia, mortality, chronic kidney disease, inflammation

## Abstract

**Background/Objectives**: Malnutrition, muscle wasting, and fluid overload are highly prevalent in patients undergoing maintenance hemodialysis (HD) and may contribute to increased mortality risk. However, the combined impact of these factors has not been fully elucidated. **Methods**: In this multicenter prospective cohort study, we enrolled 368 patients in maintenance HD at four dialysis facilities in Japan. Malnutrition was defined as moderate or higher nutritional risk using the nutritional risk index for Japanese hemodialysis patients (NRI-JH). Low muscle mass was assessed using the skeletal muscle mass index (SMI) according to the Asian Working Group for Sarcopenia 2019 (AWGS 2019), and elevated *N*-terminal pro-B-type natriuretic peptide (NT-proBNP) was defined as values in the top quartile (≥7650 pg/mL). Mortality risk was analyzed using Cox proportional hazards models. Associations with inflammation, assessed by *C*-reactive protein (CRP), were also explored. **Results**: Over a three-year follow-up period, 52 deaths occurred. Malnutrition, low muscle mass, and elevated NT-proBNP were each independently associated with increased all-cause mortality (HR: 4.98, 3.25, and 5.45, respectively). Patients with multiple concurrent risk factors had significantly worse survival. Although CRP was positively associated with these risk factors, it was not an independent predictor of mortality. **Conclusions**: Malnutrition, low muscle mass, and elevated NT-proBNP are independent and additive risk factors for mortality in HD patients. These findings highlight the need for integrated assessment and management strategies to improve prognoses in this high-risk population.

## 1. Introduction

In patients with end-stage kidney disease, hemodialysis is essential for sustaining life; however, improving long-term prognoses and preventing systemic complications remain major challenges. Hemodialysis patients are particularly vulnerable to nutritional disorders, including sarcopenia and malnutrition, which have been strongly associated with an increased risk of mortality, particularly from cardiovascular disease (CVD) [1,2]. The coexistence of chronic heart failure and fluid overload can further worsen nutritional deficits and accelerate muscle mass loss, and vice versa. These relationships further complicate the clinical challenges faced by elderly dialysis patients. This interplay of malnutrition, inflammation, and fluid overload has been conceptualized as malnutrition–inflammation–fluid-overload (MIFO) syndrome, which perpetuates a self-reinforcing cycle of deterioration [3].

Progression through the stages of chronic kidney disease leads to gradual sodium retention, resulting in the release of compensatory natriuretic peptides in response to increased stress and stretching of the left ventricular wall. *N*-terminal pro-B-type natriuretic peptide (NT-proBNP) is a well-established biomarker of cardiac stress, and elevated levels in hemodialysis patients have been associated with an increased risk of cardiovascular events and mortality [4]. NT-proBNP, although originally established as a biomarker of cardiac stress, has also been implicated in the broader pathophysiological processes observed in hemodialysis patients, including fluid overload, malnutrition, and inflammation [5,6,7]. Furthermore, inflammatory cytokines are known to stimulate NT-proBNP secretion [8]. In the context of protein-energy wasting (PEW), chronic inflammation resulting from increased muscle catabolism may contribute to both myocardial injury and the deterioration of nutritional status. Consequently, these changes can lead to further elevations in NT-proBNP levels [9]. Additionally, we speculate that thinner dialysis patients may have a reduced reserve capacity for fluid overload. Therefore, NT-proBNP may serve as a useful biomarker reflecting the combined burden of malnutrition, inflammation, and fluid overload.

However, the combined impact of malnutrition, low muscle mass, and elevated NT-proBNP levels on mortality risk remains insufficiently studied. In particular, there are limited clinical data on how the overlapping presence of these risk factors amplifies their prognostic impact.

We hypothesized that malnutrition, as assessed to be of moderate or high nutritional risk by the nutritional risk index for Japanese hemodialysis patients (NRI-JH); low muscle mass, defined according to the Asian Working Group for Sarcopenia 2019 (AWGS 2019) [10]; and elevated levels of NT-proBNP are each independently associated with increased mortality among hemodialysis patients, and that the presence of multiple concurrent risk factors would further worsen prognoses. Therefore, in this multicenter prospective study, we aimed to evaluate the individual and combined effects of these three factors on all-cause mortality and to examine their relationship with inflammation, as measured by *C*-reactive protein (CRP).

## 2. Materials and Methods

### 2.1. Study Design and Participants

Patients were considered eligible for inclusion if they were 20 years of age or older, had been receiving maintenance hemodialysis for at least 90 days, and had maintained a stable dialysis prescription for a minimum of 30 days prior to enrollment. Individuals were excluded if they had experienced coronary or valvular interventions, myocardial infarction, or hospitalization for unscheduled dialysis due to heart failure within the previous six months, or if they had echocardiographic evidence of a left ventricular ejection fraction (LVEF) below 40%. In addition, patients with pacemakers, artificial joints, or mechanical heart valves, as well as those who were pregnant or had major amputations, advanced malignancies, or dementia, were not eligible to participate.

**Figure 1:**
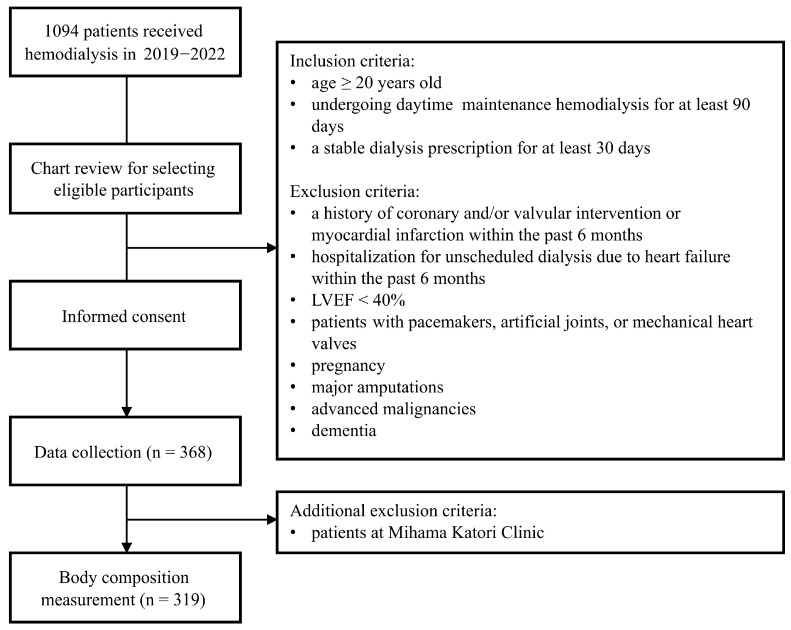
Flowchart of patient selection and inclusion/exclusion criteria.

Baseline anthropometric and laboratory data were collected from the 368 patients who met the inclusion and exclusion criteria and provided informed consent. During the subsequent body composition assessment phase, skeletal muscle mass index (SMI) measurements were not performed at the Mihama Katori Clinic due to staffing constraints. After accounting for these exclusions, 319 patients were eligible for body composition analysis. Mortality risk analyses were conducted in this subset of patients for whom SMI data were available (Figure 1).

This study was approved by the Ethics Committee of Toho University Sakura Medical Center in Tokyo, Japan (Approval Nos. S24012, 2 August 2024; S21073, 26 April 2022; and S18086, 27 December 2018), and was conducted in accordance with the principles of the Declaration of Helsinki. Written informed consent was obtained from all participants prior to enrollment.

### 2.2. Data Collection

The following baseline characteristics were collected: age, sex, and anthropometric measurements. Standard laboratory parameters were obtained during the long-interval hemodialysis session at the beginning of each month, including serum albumin, sodium, potassium, chloride, calcium, phosphorus, triglycerides, total cholesterol, low-density lipoprotein cholesterol (LDL-C), high-density lipoprotein cholesterol (HDL-C), uric acid, blood urea nitrogen, serum creatinine, intact parathyroid hormone (intact PTH), β_2_-microglobulin (β_2_MG), CRP, and hemoglobin. Pre-dialysis *N*-terminal pro-B-type natriuretic peptide (NT-proBNP) levels were measured using an electrochemiluminescence immunoassay system (Cobas 8000 e801 module; Roche Diagnostics K.K., Tokyo, Japan). The nutritional risk index for Japanese hemodialysis patients (NRI-JH) is a tool designed to assess nutritional risk in Japanese patients undergoing hemodialysis. It classifies risk levels based on serum albumin, total cholesterol, serum creatinine, and body mass index (BMI) (Table 1) [11]. Malnutrition was defined as having a moderate or high nutritional risk according to the nutritional risk index for Japanese hemodialysis patients (NRI-JH). SMI was calculated as appendicular muscle mass divided by height squared (kg/m^2^) [10]. Appendicular muscle mass was assessed in the supine position after dialysis using multi-frequency bioimpedance analysis (MF-BIA). Body composition measurements were performed using the InBody S10^®^ system (InBody Co., Ltd., Seoul, Republic of Korea; https://inbodyusa.com/, accessed on 1 November 2019). Low muscle mass was defined as skeletal muscle mass index (SMI) of <7.0 kg/m^2^ in men and <5.7 kg/m^2^ in women [10].

### 2.3. Statistical Analysis

Statistical analyses were performed using JMP Pro (version 17.0; SAS Institute Inc., Cary, NC, USA). For comparisons of continuous variables, the Welch *t*-test or Mann–Whitney U-test was used for two-group comparisons, while the Welch ANOVA or Kruskal–Wallis test was applied for comparisons between three or more groups. Fisher’s exact test was used to compare categorical variables.

Survival analysis was conducted using the Kaplan–Meier method to generate survival curves, with intergroup comparisons performed using the log-rank test. The Cox proportional hazards model was employed to estimate hazard ratios for each covariate. Statistical significance was defined as a *p*-value < 0.05.

## 3. Results

### 3.1. Population Characteristics

The demographic and clinical characteristics of the study participants (261 men and 107 women; mean age, 65 ± 12 years) are presented according to NRI-JH risk groups in Table 2. The number (percentage) of patients classified as low-, moderate-, and high-risk was 287 (77.8%), 56 (15.2%), and 25 (6.8%), respectively. Patients with higher NRI-JH risk groups tended to have lower BMIs, as well as lower serum albumin, sodium, potassium, calcium, phosphorus, triglyceride, total cholesterol, LDL-C, uric acid, blood urea nitrogen, and creatinine levels. Additionally, higher-risk NRI-JH groups were associated with lower hemoglobin and higher NT-proBNP levels. The percentage of patients aged 75 and older gradually increased according to the NRI-JH group: low-risk, moderate-risk, and high-risk. A total of 319 patients underwent body composition analysis. The body composition values by NRI-JH risk group are shown in Supplemental Table S1. Patients in the higher-risk NRI-JH groups tended to have lower total body water and intracellular water with decreased muscle mass. Of these patients, 101 (31.6%) were identified as having low muscle mass. Patients with low muscle mass were generally older, more likely to be men, and had higher NT-proBNP levels. Additionally, they exhibited lower BMIs and serum albumin, potassium, phosphorus, triglycerides, HDL-C, uric acid, and creatinine levels (Supplemental Table S2). Then, we showed population characteristics according to the quartiles of NT-proBNP levels (Supplemental Table S3). Patients with the risk factors of being in the moderate-to-high-risk NRI-JH groups, having low muscle mass, and having NT-proBNP levels in the top quartile tended to be older and male and have lower in BMIs and serum albumin, potassium, phosphorus, triglyceride, total cholesterol, HDL-C, uric acid, blood urea nitrogen, creatinine, and hemoglobin levels. These patients also tended to have higher CRP levels.

Population characteristics of patients from the Mihama Katori Clinic and those from the other participating clinics are presented in Supplemental Table S4. In light of these baseline differences, we subsequently examined the interrelationships between nutritional risk, muscle mass, and NT-proBNP levels, as well as their associations with patient outcomes.

### 3.2. Associations Between Malnutrition, Low Muscle Mass, and NT-ProBNP Levels

**Figure 2:**
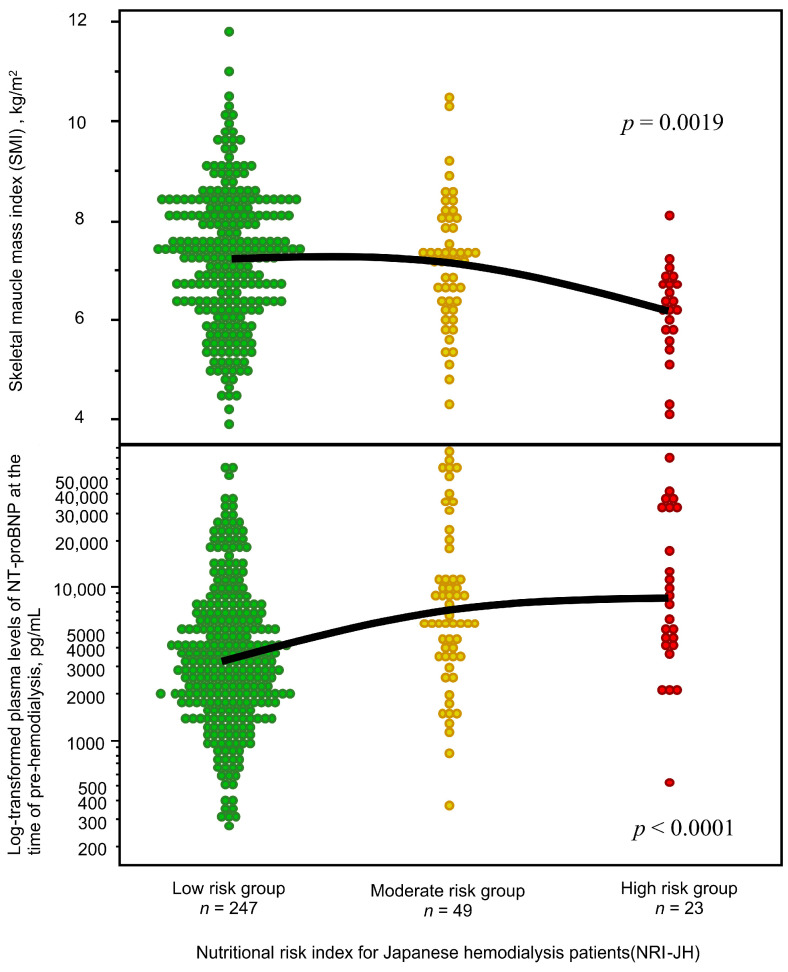
Relationships between nutritional risk index for Japanese hemodialysis patients (NRI-JH), skeletal muscle mass index, and NT-proBNP. Individual values represented by circles color-coded for each risk group (green circle: low risk group; yellow circle: moderate risk group; and red circle: high risk group) and black solid lines indicating group means.

Figure 2 illustrates the log-transformed NT-proBNP levels and SMI levels, categorized by NRI-JH risk groups. Patients in the moderate- and high-risk NRI-JH groups exhibited significantly higher NT-proBNP levels compared to those in the low-risk group. Additionally, the high-risk group demonstrated significantly lower SMI levels, whereas no substantial difference was observed between the low- and moderate-risk groups.

### 3.3. Mortality Risk Associated with Malnutrition, Low Muscle Mass, and Elevated NT-ProBNP Levels

During the three-year observation period, 52 patients died, including 15 deaths attributed to cardiovascular disease (CVD). Of these, four were due to acute myocardial infarction, four to cerebral hemorrhage, three to acute heart failure, and four to other cardiovascular causes. The remaining 37 deaths were attributed to non-CVD causes, with 13 patients succumbing to infections and 5 to malignant tumors. Additionally, 19 patients died from unknown causes or sudden death.

**Figure 3:**
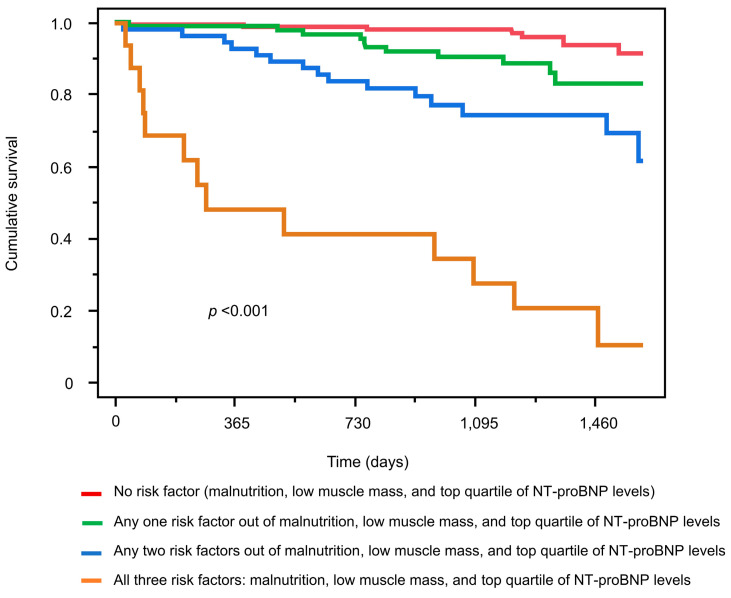
Kaplan–Meier survival curves stratified by coexistence number of malnutrition, low muscle mass, and top-quartile NT-proBNP levels in hemodialysis patients. Abbreviations: NT-proBNP, *N*-terminal-pro BNP.

**Figure 4:**
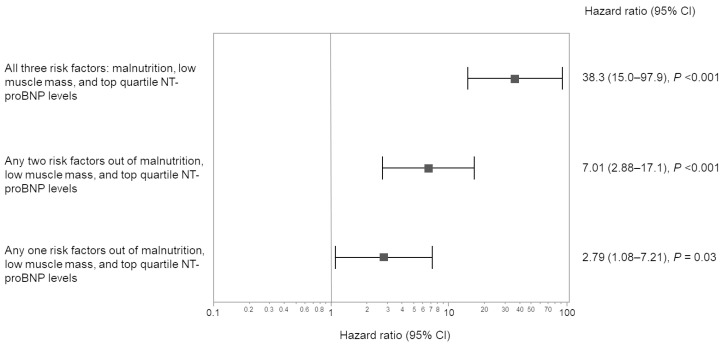
Hazard ratios for mortality by coexistence number of malnutrition, low muscle mass, and top-quartile NT-proBNP levels in hemodialysis patients. Abbreviations: NT-proBNP, *N*-terminal-pro BNP.

Among the 319 patients who underwent SMI measurement, 46 died during the observation period. Among the patients who underwent body composition analysis, the 75th percentile value of NT-proBNP was 7650 pg/mL. In the univariate analysis, Cox proportional hazards analysis revealed that moderate-to-high-risk NRI-JH groups, low muscle mass, and NT-proBNP levels in the top quartile were significantly associated with increased mortality risk, with hazard ratios of 4.98 (vs. the low-risk group), 3.25, and 5.45, respectively. These associations remained significant after adjustment for other clinical variables, indicating that each factor served as an independent predictor of mortality (Table 3). This relationship was similar when age was divided into 65 years and older or 75 years and older (Supplemental Tables S5 and S6). Additionally, 166 patients had at least one risk factor, including being in the moderate- and high-risk NRI-JH groups, having low muscle mass, or having NT-proBNP levels in the top quartile. When patients were stratified into four groups based on the number of risk factors, survival curves demonstrated significant differences between the groups (Figure 3). The mortality rate per 100 person-years increased with the number of risk factors: 1.36 in the group with no risk factor (*n* = 153; reference group), 3.78 in the group with any one risk factor (*n* = 93), 9.51 in the group with any two risk factors (*n* = 58), and 53.2 in the group with all three risk factors (*n* = 15). Eventually, the hazard ratios in these groups with any one, any two, and all three risk factors were 2.79, 7.01, and 38.3, respectively (Figure 4).

**Figure 5:**
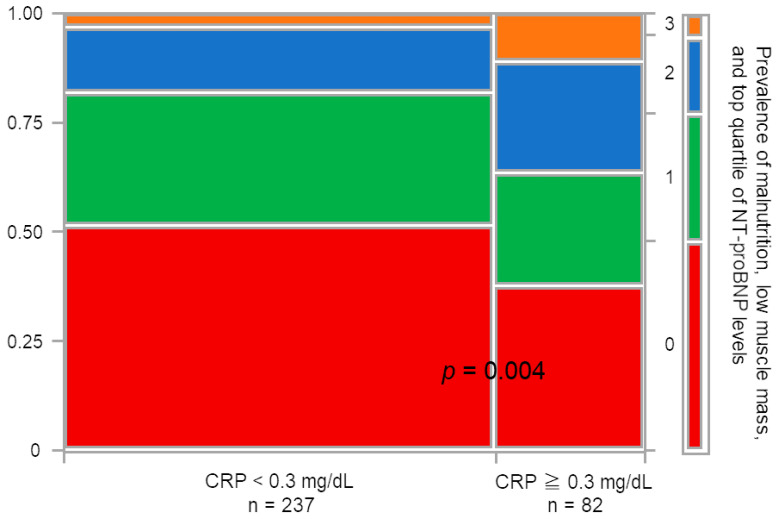
Cumulative prevalence of malnutrition, low muscle mass, and elevated NT-proBNP levels stratified by CRP category (<0.3 mg/dL vs. ≥0.3 mg/dL). Abbreviations: NT-proBNP, *N*-terminal-pro BNP.

Further analysis using a Cox proportional hazards model identified top-quartile NT-proBNP levels, low muscle mass, and moderate- and high-risk NRI-JH groups as significant independent risk factors across all models. Among the 319 patients, 82 had CRP levels ≥ 0.3 mg/dL. The high-CRP group exhibited a significantly greater accumulation of risk factors compared to the low-CRP group, as determined by Fisher’s exact test (*p* = 0.004). However, despite this higher prevalence of risk factors, elevated CRP was not identified as an independent predictor of mortality in the Cox proportional hazards model (Table 3 and Figure 5).

## 4. Discussion

This study demonstrated that elevated NT-proBNP levels were associated with both malnutrition and low muscle mass, and that each of these factors—malnutrition, low muscle mass, and elevated NT-proBNP—served as an independent predictor of mortality. Moreover, the presence of multiple concurrent risk factors was associated with a markedly increased risk of death. In contrast, although patients in the high-CRP group tended to exhibit a greater burden of risk factors, CRP itself was not identified as an independent predictor of mortality. This may be partly attributable to the limited sample size; alternatively, CRP may act as a nonspecific marker of systemic inflammation—reflecting both acute and chronic inflammatory states—rather than directly influencing mortality risk. Furthermore, simultaneous adjustment for NT-proBNP, SMI levels, and nutritional status may have attenuated the relative contribution of CRP, resulting in a hazard ratio below 1.0. CRP may not independently influence mortality risk when nutritional status and cardiac stress are accounted for, suggesting that its elevation reflects an underlying inflammatory state rather than serving as a direct driver of mortality.

Extensive research has examined the interrelationships between sarcopenia, heart failure, and malnutrition within the framework of chronic inflammation [12,13], as well as their individual impact on mortality risk [14,15]. Moreover, NT-proBNP levels are known to be positively correlated with volume overload [16,17]. However, few studies have systematically investigated the cumulative impact of these factors when present simultaneously. The findings of this study are consistent with previous reports, suggesting that clinical deterioration is exacerbated when multiple risk factors coexist.

The pathophysiological mechanisms underlying these outcomes likely involve systemic metabolic disturbances mediated by chronic inflammation and oxidative stress. Chronic inflammation, often associated with aging and arteriosclerosis, promotes catabolism and suppresses anabolism via inflammatory cytokines. In skeletal muscle, this leads to accelerated protein degradation, impaired protein synthesis, and progressive muscle wasting. In the heart, oxidative stress and mitochondrial dysfunction contribute to cardiomyocyte injury and progressive cardiac impairment. The progression of malnutrition and sarcopenia is further exacerbated by heightened catabolism and suppressed anabolism, ultimately resulting in a decline in dry weight. This weight loss induces fluid overload, creating a discrepancy between post-dialysis weight and actual fluid balance, which perpetuates a self-reinforcing cycle of nutritional decline and muscle wasting. Persistent fluid overload imposes chronic hemodynamic stress on the heart, exacerbating oxidative stress and myocardial inflammation. Additionally, muscle depletion, itself an inflammatory process, contributes to a progressive cycle of deterioration. This phenomenon, recognized as malnutrition–inflammation–fluid-overload (MIFO) syndrome, has been increasingly identified as a hallmark of dialysis-related complications.

The findings of this study highlight the critical importance of nutritional optimization and body weight maintenance in hemodialysis patients. A meta-analysis evaluating the effects of exercise therapy and protein intake on inflammatory response modulation in older adults found that combining exercise therapy with protein intake led to greater reductions in inflammatory markers compared to exercise therapy alone [18]. Likewise, in hemodialysis patients, intradialytic exercise therapy has been shown to attenuate inflammation, maintain physical and social function, and enhance both overall survival and healthy life expectancy [19,20,21]. In conclusion, our findings highlight the need for comprehensive care in hemodialysis patients that addresses nutritional status, muscle mass preservation, and fluid management. Interventions such as nutritional support and intradialytic exercise may be important to improve survival in this vulnerable population. Moreover, because fluid overload itself may contribute to the development or worsening of malnutrition, therapeutic strategies aimed at reducing excess fluid retention could be as important as nutritional interventions in improving clinical outcomes.

This study has several limitations. First, it did not take anthropometric measurements, such as biceps circumference, which are typically used in nutritional assessments. Instead, we presented our body composition data by NRI-JH risk group in the Supplemental Table S1. Next, muscle mass assessment was restricted to SMI levels, excluding muscle strength and functional assessments. The AWGS 2019 criteria for sarcopenia require a comprehensive evaluation that includes muscle strength and functional capacity, suggesting that the definition of “low muscle mass” in this study may not fully align with sarcopenia diagnoses. Third, bioelectrical impedance analysis, used for body composition assessment, has been shown to overestimate muscle mass in the presence of fluid retention, potentially leading to measurement bias. To improve the accuracy and generalizability of future research, study designs should integrate alternative methodologies such as dual-energy X-ray absorptiometry or magnetic resonance imaging, which provide more precise assessments of muscle mass and fluid status. Fourth, due to logistical constraints, not all patients who gave consent underwent SMI measurement; those without SMI data were excluded, which could introduce bias if their nutritional or clinical profiles differed. However, there were no significant differences in the distribution of patients with nutritional disorders or NT-proBNP levels between the Katori Clinic and the other three clinics.

## 5. Conclusions

In conclusion, malnutrition, low muscle mass, and elevated NT-proBNP levels were independent predictors of mortality in patients undergoing maintenance hemodialysis. Notably, the coexistence of these risk factors was associated with a markedly increased risk of mortality, suggesting that the adverse effects of malnutrition, muscle wasting, volume overload, or cardiac overload may be additive or synergistic in nature. These findings highlight the importance of routine screening and integrated interventions targeting nutritional status, muscle health, and fluid volume management in high-risk hemodialysis patients. Further studies are needed in the future to determine whether proactive correction of these abnormalities will improve survival.

## Figures and Tables

**Table 1:** NRI-JH scoring process.

Risk Marker	Score Value
Body mass index	
<20 kg/m^2^	3
Serum albumin	
<3.7 g/dL (Age < 65 years); <3.5 g/dL (Age ≥ 65 years)	4
Serum creatinine	
Women <9.7 mg/dL (Age < 65 years); <8.0 mg/dL (Age ≥ 65 years) Men <11.6 mg/dL (Age < 65 years); <9.7 mg/dL (Age ≥ 65 years)	4
Serum total cholesterol	
<130 mg/dL	1
≥220 mg/dL	2
Categorization based on the total score: 0–7 points: low risk, 8–10 points: moderate risk, 11 points or more: high risk	

Abbreviations: BMI: body mass index, NRI-JH: nutritional risk index for Japanese hemodialysis patients.

**Table 2:** Population characteristics according to NRI-JH.

Patient Characteristics	NRI-JH			*p*
	Low Risk (*n* = 287)	Moderate Risk (*n* = 56)	High Risk (*n* = 25)	
Age, years	67 (56–74)	64.5 (59–75)	76 (56–82)	0.06
Age < 65, *n* (%)	107 (37.3)	28 (50.0)	8 (32.0)	
Age 65–74, *n* (%)	120 (41.8)	13 (23.2)	3 (12.0)	<0.001
Age ≥ 75, *n* (%)	60 (20.8)	15 (26.8)	14 (56.0)	
Men, *n* (%)	201 (70.0)	42 (75.0)	18 (72.0)	0.75
Diabetes, *n* (%)	129 (45.0)	32 (57.1)	7 (28.0)	0.046
Body mass index, kg/m^2^	22.7 (20.2–25.6)	22.1 (20.8–25.0)	18.3 (17.0–19.3)	<0.001
Serum albumin, g/dL	3.6 (3.5–3.8)	3.4 (3.2–3.5)	3.3 (3.1–3.4)	<0.001
Serum sodium, mEq/L	139 (138–141)	139 (137–141)	138 (137–139)	0.047
Serum potassium, mEq/L	4.9 (4.4–5.4)	4.7 (4.2–5.0)	4.7 (4.2–5.2)	0.028
Serum chloride, mEq/L	103 (101–105)	104 (102–106)	103 (102–106)	0.48
Serum calcium, mg/dL	8.7 (8.3–9.0)	8.6 (8.2–8.9)	8.3 (8.0–8.7)	0.011
Serum phosphorus, mg/dL	5.6 (5.0–6.4)	5.1 (4.4–5.7)	5.4 (4.7–6.5)	<0.001
Triglyceride, mg/dL	105 (75–154)	91 (67–127)	64 (51–87)	<0.001
Total cholesterol, mg/dL	165 (143–193)	152 (130–185)	159 (130–191)	0.040
LDL-C, mg/dL	87 (71–107)	77.5 (62–104)	75 (61–102)	0.049
HDL-C, mg/dL	50 (39–60)	45 (37–57)	54 (42–70)	0.11
Uric acid, mg/dL	7.9 (7.1–8.6)	6.9 (6.2–8.0)	6.9 (6.5–8.0)	<0.001
Blood urea nitrogen, mg/dL	60.8 (52.2–71.4)	49.6 (43.9–56.8)	48.9 (45.5–59.0)	<0.001
Serum creatinine, mg/dL	10.7 (9.4–12.3)	8.8 (7.7–9.8)	8.6 (7.4–9.0)	<0.001
Intact PTH, pg/mL	154 (102–223)	157 (99–197)	138 (79–188)	0.41
β_2_MG, mg/L	26.0 (23.4–29.2)	27.0 (22.6–29.7)	26.6 (25.4–34)	0.27
*C*-reactive protein, mg/dL	0.1 (0–0.3)	0.1 (0.1–0.5)	0.3 (0–0.6)	0.19
Hemoglobin, g/dL	11.3 (10.7–11.9)	11.0 (10.5–11.5)	10.6 (9.8–11.4)	0.002
NT-proBNP, pg/mL	3000 (1610–6610)	5900 (3478–11,075)	7650 (4045–32,450)	<0.001

**Table 3:** Independent risk factors and hazard ratios for mortality (Cox regression analysis).

Variables	Univariate Analysis			Multivariate Analysis		
	HR	95%CI	*p*	HR	95%CI	*p*
Age	1.06	(1.04–1.09)	<0.001	1.05	(1.02–1.08)	<0.001
Diabetes	1.66	(0.93–2.98)	0.09	1.48	(0.80–2.77)	0.21
Men	2.15	(1.00–4.62)	0.049	2.15	(0.95–4.86)	0.07
CRP ≥ 0.3 mg/dL	1.33	(0.71–2.50	0.37	0.74	(0.38–1.45)	0.38
Moderate to high risk by NRI-JH	4.98	(2.79–8.91)	<0.001	3.52	(1.92–6.44)	<0.001
Low muscle mass	3.25	(1.81–5.82)	<0.001	2.63	(1.42–4.86)	0.002
Top-quartile NT-proBNP levels	5.45	(3.02–9.88)	<0.001	3.40	(1.81–6.38)	<0.001

Abbreviations: NRI-JH, nutritional risk index for Japanese hemodialysis patients; NT-proBNP, *N*-terminal-pro BNP.

## Data Availability

The data presented in this study are available from the corresponding author upon reasonable request.

## Supplementary Materials

The following supporting information can be downloaded at: https://www.mdpi.com/article/10.3390/nu17111896/s1, Table S1: body composition according to NRI-JH; Table S2: Population characteristics according to low muscle mass; Table S3: Population characteristics according to quartile of NT-proBNP; Table S4: Population characteristics in Katori clinic and the other 3 clinics; Table S5: Independent risk factors and hazard ratios for mortality using valuables of age ≥ 65 years or not; Table S6: Independent risk factors and hazard ratios for mortality using valuables of age ≥ 75 years or not.
