# Supplementary material for: Association Between Malnutrition, Low Muscle Mass, Elevated NT-ProBNP Levels, and Mortality in Hemodialysis Patients
Source: Nutrients. 2025 May 31;17(11):1896. doi: 10.3390/nu17111896 (PMC12157709; doi:10.3390/nu17111896)
Supplement: Supplementary file 1 [file nutrients-17-01896-s001.zip › Supplemental table S1. body composition according to NRI-JH.pdf]

Suppliment Table S1. body composition according to NRI-JH

Patient Characteristics	NRI-JH			*p*
	Low risk (n = 247)	Moderate risk (n = 50)	High risk (n = 22)	
Body weight, kg	60.4 (51.6–69.8)	59.5 (54.1–66.6)	48.0 (43.9–51.5)	<0.001
Total body water, kg	33.5 (18.3–60.9)	34.0 (20–47.2)	30.0 (20–41.6)	0.044
%Total body water, %	55 (50–59)	55 (50–61)	62 (57–66)	<0.001
Intracellular body water, kg	20.4 (16.5–23.7)	20.6 (17.1–22.7)	17.9 (16.6–19.2)	0.022
%Intracellular body water, %	33.4 (30.9–36.0)	32.5 (30.1–36.5)	36.9 (34.1–39.3)	<0.001
Extracellular body water, kg	12.9 (10.5–14.8)	13.4 (11.5–14.6)	11.8 (10.5–13.2)	0.101
%Extracellular body water, %	21.1 (19.4–23.1)	22.3 (19.4–23.8)	25.0 (22.7–26.6)	<0.001
Protein, kg	8.8 (7.2–10.3)	8.9 (7.3–9.9)	7.75 (7.1–8.3)	0.022
%Protein, %	14.4 (13.3–15.6)	14.1 (13.0–15.9)	15.9 (14.8–17.1)	<0.001
Mineral, kg	3.04 (2.56–3.52)	3.08 (2.67–3.52)	2.71 (2.55–2.99)	0.054
%Mineral, %	5.1 (4.6–5.5)	4.95 (4.6–5.8)	5.8 (5.4–6.2)	<0.001
Fat, kg	14.9 (10.8–21.6)	14.2 (10.5–19.9)	7.9 (4.7–11.0)	<0.001
%Fat, %	26.0 (19.8–31.5)	26.1 (17.5–32.5)	16.6 (10.9–22.7)	<0.001
Lean body mass, kg	45.2 (36.9-52.8)	46.1 (38.4-50.9)	40.4 (36.6-43.2)	0.039
Skeletal muscle mass, kg	24.6 (19.6-29)	24.9 (20.2-27.7)	21.4 (19.3-23.2)	0.022
Skeletal muscle mass index, kg/m^2	7.3 (6.3-8.2)	7.2 (6.3-8.0)	6.3 (5.7-6.7)	<0.001

Note: Body composition was assessed using multi-frequency bioelectrical impedance analysis (MF-BIA) based on a four-compartment model. This model estimates body composition by dividing total body mass into four components: fat mass, protein mass, mineral content, and total body water. Total body water consists of both intracellular and extracellular compartments, and lean body mass is calculated as the sum of protein, minerals, and total body water. Measurements include both absolute values (kg) and percentages (%) relative to total body weight.
