# Supplementary material for: Association Between Malnutrition, Low Muscle Mass, Elevated NT-ProBNP Levels, and Mortality in Hemodialysis Patients
Source: Nutrients. 2025 May 31;17(11):1896. doi: 10.3390/nu17111896 (PMC12157709; doi:10.3390/nu17111896)
Supplement: Supplementary file 1 [file nutrients-17-01896-s001.zip › Supplemental table S2. Population characteristics according to low muscle mass.pdf]

Supplemental table S2. Population characteristics according to low muscle mass

Patient Characteristics	Low muscle mass		*p*
	absence (n = 218)	presence (n = 101)	
Age, years	65 (53.75–73)	72 (65–77)	<0.001
Men, n (%)	162 (74.3)	61 (60.4)	0.013
Diabetes, n (%)	103 (47.2)	40 (39.6)	0.23
Body mass index, kg/m^2	23.25 (20.975–26.1)	19.6 (18.05–21.4)	<0.001
Serum albumin, g/dL	3.6 (3.5–3.8)	3.4 (3.3–3.6)	<0.001
Serum sodium, mEq/L	139 (137–140)	139 (137–141)	0.69
Serum potassium, mEq/L	4.8 (4.4–5.4)	4.8 (4.3–5.1)	0.005
Serum chloride, mEq/L	103 (102–105)	104 (102–106)	0.19
Serum calcium, mg/dL	8.7 (8.3–9)	8.5 (8.2–8.9)	0.12
Serum phosphorus, mg/dL	5.6 (5–6.5)	5.3 (4.8–6.1)	0.032
Triglyceride, mg/dL	107.5 (72–155.25)	86 (61.5–113.5)	<0.001
Total cholesterol, mg/dL	162 (140–185.25)	166 (140.5–197.5)	0.27
LDL-C, mg/dL	85 (67.75–106)	83 (70–109)	0.51
HDL-C, mg/dL	47 (38–59)	53 (42.5–65.5)	0.006
Uric acid, mg/dL	7.8 (6.875–8.6)	7.3 (6.7–8.15)	0.002
Blood urea nitrogen, mg/dL	59 (49.85–68)	55.1 (46.9–69.8)	0.37
Serum creatinine, mg/dL	10.62 (9.17–12.38)	9.1 (8.09–10.5)	<0.001
Intact PTH, pg/mL	159 (106.75–231.25)	144 (85.5–203)	0.14
β_2MG, mg/L	26 (23.2–29)	26.4 (23.25–29.9)	0.41
C-reactive protein, mg/dL	0.12 (0.05–0.272)	0.16 (0.054–0.374)	0.27
Hemoglobin, g/dL	11.2 (10.7–12.1)	11.2 (10.5–11.7)	0.06
NT-proBNP, pg/mL	3435 (1698–6713)	4630 (2060–9245)	0.025

Abbreviations: LDL-C, low-density lipoprotein cholesterol; HDL-C, high-density lipoprotein cholesterol; Intact PTH, Intact parathyroid hormone; NT-proBNP, N-terminal-pro BNP
