# Supplementary material for: Association Between Malnutrition, Low Muscle Mass, Elevated NT-ProBNP Levels, and Mortality in Hemodialysis Patients
Source: Nutrients. 2025 May 31;17(11):1896. doi: 10.3390/nu17111896 (PMC12157709; doi:10.3390/nu17111896)
Supplement: Supplementary file 1 [file nutrients-17-01896-s001.zip › Supplemental table S3. Population characteristics according to quartile of NT-proBNP.pdf]

Supplemental table S3. Population characteristics according to quartile of NT-proBNP

Patient Characteristics	NT-proBNP (pg/dL)				*p*
	**Quartile 1 <1795 (n =92)**	**Quartile 2 1795-3590 (n = 92)**	**Quartile 3 3590-7718 (n = 92)**	**Quartile 4 >7718 (n = 92)**	
Age, years	64 (50–71)	67 (54–73)	70.5 (57–77)	70 (63–76)	<0.001
Men, n (%)	73 (79.3)	60 (65.2)	54 (58.7)	74 (80.4)	0.002
Body mass index, kg/m^2	24.25 (20.6–27.1)	22.45 (20.1–24.7)	21.2 (19.4–25.4)	21.25 (19.3–24.0)	0.006
Diabetes, n (%)	41 (44.6)	42 (45.7)	36 (39.1)	49 (53.3)	0.003
Serum albumin, g/dL	3.7 (3.5–3.9)	3.6 (3.5–3.9)	3.55 (3.3–3.7)	3.5 (3.2–3.6)	0.29
Serum sodium, mEq/L	139 (138–141)	139 (137–141)	139 (137–140)	139 (137–140)	<0.001
Serum potassium, mEq/L	4.75 (4.2–5.2)	4.95 (4.5–5.5)	4.85 (4.5–5.3)	4.8 (4.4–5.2)	0.10
Serum chloride, mEq/L	103 (101–105)	103 (102–105)	104 (102–106)	103 (101–106)	0.09
Serum calcium, mg/dL	8.7 (8.3–8.9)	8.7 (8.3–9)	8.5 (8.2–8.9)	8.7 (8.2–8.9)	0.51
Serum phosphorus, mg/dL	5.55 (4.8–6.2)	5.6 (5.2–6.4)	5.4 (4.9–6.4)	5.5 (4.7–6.2)	0.52
Triglyceride, mg/dL	130 (78–187)	104 (66–147)	97.5 (68–138)	86 (61–110)	0.18
Total cholesterol, mg/dL	169 (149–198)	165 (140–198)	161.5 (140–188)	154 (131–176)	0.004
LDL-C, mg/dL	90 (74–109)	82 (71–107)	83.5 (65–104)	78.5 (66–97)	0.046
HDL-C, mg/dL	45 (37–55)	51.5 (41–64)	51 (41–64)	47.5 (38–58)	0.009
Uric acid, mg/dL	8.1 (7.3–9.2)	7.8 (7.1–8.5)	7.6 (6.8–8.3)	7.15 (6.4–8.1)	<0.001
Blood urea nitrogen, mg/dL	59.7 (50–71.2)	60.65 (53.4–72.9)	59 (49.7–66.6)	52.6 (45–62.1)	<0.001
Serum creatinine, mg/dL	11.21 (9.09–12.82)	10.67 (9.4–11.82)	9.89 (8.38–11.08)	9.395 (8.48–10.7)	<0.001
Intact PTH, pg/mL	164.5 (109–230)	137 (84–194)	168 (114–231)	159 (91–217)	0.06

β_2MG, mg/L	26 (22.3–28.5)	26.5 (23–29.9)	25.3 (23–28.6)	27.2 (25–30)	0.016
C-reactive protein, mg/dL	0.14 (0.05–0.22)	0.07 (0.04–0.20)	0.10 (0.04–0.30)	0.15 (0.07–0.40)	0.001
Hemoglobin, g/dL	11.4 (10.7–12.1)	11.3 (10.8–11.9)	11 (10.6–11.5)	10.9 (10.4–11.6)	0.002

Abbreviations: LDL-C, low-density lipoprotein cholesterol; HDL-C, high-density lipoprotein cholesterol; Intact PTH, Intact parathyroid hormone; NT-proBNP, N-terminal-pro BNP
