# Supplementary material for: Association Between Malnutrition, Low Muscle Mass, Elevated NT-ProBNP Levels, and Mortality in Hemodialysis Patients
Source: Nutrients. 2025 May 31;17(11):1896. doi: 10.3390/nu17111896 (PMC12157709; doi:10.3390/nu17111896)
Supplement: Supplementary file 1 [file nutrients-17-01896-s001.zip › Supplemental table S4. Population characteristics in Katori clinic and the other 3 clinics.pdf]

Supplemental table S4. Population characteristics in Katori clinic and the other 3 clinics

Patient Characteristics	Katori clinic (n = 49)	The others 3 clinics (n = 319)	*p*
Age, years	66 (52–72)	67 (56–74)	0.28
Men, n (%)	38 (77.6)	223 (69.9)	0.31
Diabetes, n (%)	25 (51.0)	143 (44.8)	0.44
Body mass index, kg/m^2	24.2 (20.4–26)	22 (19.8–25.3)	0.027
Serum albumin, g/dL	3.6 (3.4–3.9)	3.6 (3.4–3.7)	0.08
Serum sodium, mEq/L	140 (137–141)	139 (137–140)	0.29
Serum potassium, mEq/L	4.7 (4.4–5.2)	4.8 (4.4–5.3)	0.57
Serum chloride, mEq/L	103 (100–104)	103 (102–106)	0.07
Serum calcium, mg/dL	8.6 (8.3–8.8)	8.6 (8.3–8.9)	0.38
Serum phosphorus, mg/dL	5.6 (5.1–6.1)	5.6 (4.9–6.3)	0.99
Triglyceride, mg/dL	120 (71–161)	98 (68–144)	0.15
Total cholesterol, mg/dL	162 (133–193)	163 (140–189)	0.77
LDL-C, mg/dL	85 (69–99)	85 (68–106)	0.98
HDL-C, mg/dL	47 (34–54)	49 (40–61)	0.032
Uric acid, mg/dL	8.1 (7.5–8.7)	7.6 (6.8–8.4)	0.005
Blood urea nitrogen, mg/dL	56.4 (47.3–70.3)	58 (48–68.2)	0.92
Serum creatinine, mg/dL	10.57 (8.82–11.6)	9.99 (8.77–11.78)	0.35
Intact PTH, pg/mL	140 (98–202)	156 (102–220)	0.15
β_2MG, mg/L	26.8 (23.8–29.7)	26.15 (23.2–29.1)	0.31
C-reactive protein, mg/dL	0.075 (0.037–0.166)	0.13 (0.05–0.302)	0.033
Hemoglobin, g/dL	11.2 (10.7–11.6)	11.2 (10.6–11.9)	0.70
NT-proBNP, pg/mL	3210 (1220–9540)	3690 (1850–7650)	0.43
NRI-JH Risk, n (%)			0.794
Mild	40 (81.6)	247 (77.4)	
Moderate	6 (12.2)	50 (15.7)	
High	3 (6.1)	22 (6.9)	

Abbreviations: LDL-C, low-density lipoprotein cholesterol; HDL-C, high-density lipoprotein cholesterol; Intact PTH, Intact parathyroid hormone; NT-proBNP, N-terminal-pro BNP
