# Supplementary material for: Association Between Malnutrition, Low Muscle Mass, Elevated NT-ProBNP Levels, and Mortality in Hemodialysis Patients
Source: Nutrients. 2025 May 31;17(11):1896. doi: 10.3390/nu17111896 (PMC12157709; doi:10.3390/nu17111896)
Supplement: Supplementary file 1 [file nutrients-17-01896-s001.zip › Supplemental table S5. Independent risk factors and hazard ratios for mortality (age ¿R65 and age ¿R75).pdf]

Supplemental table S5. Independent risk factors and hazard ratios for mortality using age ≧65 years or not.

Variables	Univariate analysis			Multivariate analysis		
	HR	95%CI	*p*	HR	95%CI	*p*
Age≧65 years	2.83	(1.50–5.79)	<0.001	2.59	(1.25–5.34)	0.010
Diabetes mellites	1.66	(0.93–2.98)	0.09	1.48	(0.79–2.75)	0.22
Men	2.15	(1.00–4.62)	0.049	1.93	(0.86–4.33)	0.11
CRP ≧ 0.3 mg/dL	1.33	(0.71–2.50	0.37	0.71	(0.37–1.41)	0.34
Moderate to high risk by NRI-JH	4.98	(2.79–8.91)	<0.001	4.08	(2.21–7.56)	<0.001
Low muscle mass	3.25	(1.81–5.82)	<0.001	2.81	(1.52–5.19)	<0.001
Top quartile of NT-proBNP levels	5.45	(3.02–9.88)	<0.001	3.81	(2.03–7.14)	<0.001
