# Supplementary material for: Association Between Malnutrition, Low Muscle Mass, Elevated NT-ProBNP Levels, and Mortality in Hemodialysis Patients
Source: Nutrients. 2025 May 31;17(11):1896. doi: 10.3390/nu17111896 (PMC12157709; doi:10.3390/nu17111896)
Supplement: Supplementary file 1 [file nutrients-17-01896-s001.zip › Supplemental table S6. Independent risk factors and hazard ratios for mortality (age ¿R65 and age ¿R75).pdf]

Supplemental table S6. Independent risk factors and hazard ratios for mortality using age ≧75 years or not.

Variables	Univariate analysis			Multivariate analysis		
	**HR**	**95%CI**	***p***	**HR**	**95%CI**	***p***
Age≧75 years	3.46	(1.98–6.00)	<0.001	2.90	(1.55–5.39)	0.007
Diabetes mellites	1.66	(0.93–2.98)	0.09	1.50	(0.81–2.77)	0.20
Men	2.15	(1.00–4.62)	0.049	2.19	(0.98–4.90)	0.057
CRP ≧ 0.3 mg/dL	1.33	(0.71–2.50	0.37	0.79	(0.40–1.54)	0.49
Moderate to high risk by NRI-JH	4.98	(2.79–8.91)	<0.001	3.28	(1.79–6.00)	<0.001
Low muscle mass	3.25	(1.81–5.82)	<0.001	2.83	(1.53–5.23)	<0.001
Top quartile of NT-proBNP levels	5.45	(3.02–9.88)	<0.001	3.87	(2.07–7.23)	<0.001
